# The Re-Election of Hospital Staffs

**Published:** 1909-10-16

**Authors:** 


### SPECIAL ARTICLE.

### THE RE-ELECTION OF HOSPITAL STAFFS.

An advetisement which has been appearing recently in the columns of some of the professional papers suggests discussion of a very old question of which little has lately been heard. The notice is one asking for applications for the post of honorary anæsthetist to one of the best-known special hospitals in London, the said post to be held for a period of five years, and the holder to be eligible for re-election at the end of that period. Now, as far as anæsthetists are concerned, the value of this provision can hardly be disputed; for if a man after five years' practice is not a competent anæsthetist he is never likely to become one; and the sooner he makes room for somebody else, the better. In fact, the term of office might very well be made three, or even two, years in the case of anæsthetists.

When once this principle is admitted—and although we do not expect universal, or even general, agreement, it must be conceded that there is much to be said in its favour—it cannot logically be limited to anæsthetists. If it is right in the public interests and in the interests of hospitals that some non-resident hospital appointments shall be liable to revision at stated intervals, then of a surety physicians and surgeons stand on a footing strictly analogous to that of anæsthetists. As matters stand, it happens only too frequently that young men of promise, whose academic achievements are at least of a high and often of a brilliant order, are elected to the out-patient staff of a hospital at a time when no one has any chance of knowing whether they do or do not possess those other qualities without which no amount of book knowledge will make a successful clinician. It is true that in the teaching hospitals the seniors have, as a rule, the advantage of having watched the careers of the candidates for any vacancy as students, residents, registrars, demonstrators, and so on; but even that gives them no real opportunity in most cases of judging accurately the undeveloped resources of character and craftsmanship latent for lack of opportunity. That this is so is easy enough to demonstrate. Let any who doubt it look up the careers, as given in the *Medical Directory*, of the medical and surgical staff of any leading hospital with a medical school. It will be found, time and again, that one at least (and often more than one) of the most efficient and successful of the visiting staff have been educated at some other hospital, where their abilities and promise have not been recognised, or have, at any rate, been considered inferior to those of some other person whose performances have turned out a comparative failure. A dozen examples could be mentioned offhand, were it necessary or politic to do so, in London alone at the present day, and a very little research would compile a list such as would certainly astonish any who have not considered the matter.

### The Personal Factor.

An instance of a curious mistake in judgment, though of a different kind, could be quoted in this connection. It was fairly common knowledge about twenty years ago that at a well-known teaching hospital the selection of a candidate to fill a vacancy on the junior visiting staff was decided practically by two of the seniors; and also that those two seniors voted for a certain candidate because they were under the impression that, excellent as his qualifications were, he was not likely ever to prove a dangerous competitor in private consulting practice. The sequel to this very degrading transaction was both amusing and instructive; for within five years the young man is credibly alleged to have been making an income greater than that of his two seniors put together, and it is hard to say whether he or the hospital has benefited most by the connection between them. This story, which is not in the least a caricature of the facts, is quoted to illustrate two points. The first, upon which insistence has been already laid, is that it is very difficult to judge of the future performances of a budding consultant, not only from his academical record, but even also from that more intimate association which exists between the resident staff and their seniors. The other is that personal considerations enter so largely into the election of hospital staffs that they overshadow to a very great extent the other qualifications of the candidates. It is not being argued that personal considerations can be or should be entirely neglected; such a state of affairs is impossible in the very nature of human society. Nor is it altogether desirable; for the professional staff of a hospital are in a sense a committee, if not actually a family, and the presence of one of those persons who are generally described by their friends as " impossible to work with " (and by their enemies in harsher terms) produces endless friction and much more harm than good to the institution. But it is affirmable without fear of contradiction that the present system leads inevitably to a certain amount of sycophancy, and that injustice is sometimes done to those who have refused to exhibit the necessary supply of that particularly detestable form of adaptability. Anyone whose experience as a resident has been at all prolonged, or who has watched the manœuvres to which the knowledge of an approaching vacancy on the visiting staff of a hospital may lead, must recognise and admit the truth of these generalisations.

### Hospital Failures.

We have arrived, then, at these conclusions, that for various reasons those elected to visiting hospital staffs are not, or do not always turn out to be, the best men available. We would go further, and assert that even those whose selection appears for the first few years of their career quite unimpeachable develop sometimes fads or peculiarities which render their removal from office desirable. As matters stand, the only possible chance of getting rid of a failure is by rejecting him when the rotation of vacancies brings him, as head of the assistant staff, to candidature for appointment to the senior staff. Unfortunately the rejection of a man by his hospital at this epoch would almost certainly mean his professional extinction, at least as a consultant, and this his colleagues are afraid of being thought to desire. As a rule only laches of conduct very gross from a moral or social point of view will lead to such rejection. The result is that a man once elected to the visiting staff of many hospitals is practically a fixture there until the age of sixty, or sixty-five, or whatever age is prescribed for retirement by the rules of the hospital. This system leads to the implantation of a certain number of square pegs in round holes, and although in the main it works well it is certainly capable of improvement. Instances could be multiplied of surgeons whose incapacity is painfully obvious to every onlooker, and of physicians to whose care not one of their colleagues would willingly entrust his life. More, it happens now and then that a once brilliant intellect becomes obscured later on, and that a man is allowed, for the sake of avoiding an open scandal, to retain his position long after all the rest of the staff are convinced of his utter unfitness for it.

It may be said that these things constitute an indictment of the honesty and the honour of the cream of the medical profession, and in a sense this is so. Undoubtedly there is displayed over this question a supineness that does little credit to the consultants. But it must also be remembered that the position in circumstances such as some of those that have been indicated is a difficult one. To give a man a fair chance, when he seems not to be displaying the technical ability expected of him, is the natural instinct of those who have elected him to office; and so matters drag on until any course of action would seem to be a brand of patent and abject incapacity. Moreover, motives the reverse of creditable might always, and probably often would be, imputed to any who should attempt to replace a mediocre colleague by one more gifted. But at the same time it must be pointed out that there is first and foremost the public to be considered. Hospital appointments are not awarded for the convenience of the medical profession, but for the benefit of the public and of the hospital, whose interests are identical. It is a duty, and a very sacred one, that no class interest, no spirit of trades unionism (with all deference to our rulers) shall be an obstacle to the provision of the very best, and nothing but the very best, medical and surgical *personnel* for our voluntary hospitals. It were better for an inefficient consultant that a millstone were hung about his neck and he were cast into general practice than that he should continue to render services to his hospital which could be bettered by the appointment of somebody else to his place. And since no man is capable of seeing for himself and judging correctly whether this be so or not, the decision must rest with those professional colleagues who are associated with him in his work and have the proper opportunities for estimating its quality.

### Terminable Appointments.

This desirable state of things can be achieved, in our judgment, only by an alteration of the present system, whereby every appointment would be reviewed at a fixed interval of three, four, or five years. That such a proposal has disadvantages cannot be denied, though probably they are far less than has been supposed by those who favour the maintenance of the existing arrangement. That it might conceivably lead to the ostracism and extinction of some great genius years ahead of his times by a set of muddleheads such as those who openly derided Lister we do not believe to be even remotely possible. Scientific methods and the true scientific spirit are now so taught and inculcated in the preliminary education of the modern medical man that the danger of the hour is not undue conservatism in accepting the most recent researches, but undue willingness to adopt anything alleged by an enthusiastic investigator to be the latest advance, without waiting for a critical judgment from some competent tribunal. That it might lead to ill-feeling among the members of hospital staffs is equally unlikely; and even if it did, that is of small moment compared with the public weal and the advantage of patients. That insecurity of tenure might prevent originality and incline men to play the safe game is also not to be feared; for proved incapacity or moral shortcomings would be the least that would justify rejection at the quinquennial revision, and the general sense of fair play among a body of men of the standing of a hospital staff could be trusted to prevent any suspicion of jealousy or other ignoble motives becoming operative. Undoubtedly evils exist such as have been alluded to in connection with the visiting staffs of hospitals. Undoubtedly those evils can be and must be mitigated. If anyone has any suggestions to offer towards such an end, by all means let them be made public. But meanwhile the attention of the profession may very seriously be drawn to the solution provided by some scheme of review and re-election as advocated above. Reforms come best from within; if they are forced from without they are generally imperfect and sometimes unworkable. Let us therefore honestly consider whether the present system is not susceptible of improvement, remembering always that the welfare of the public and the honour of the profession come first and foremost and beyond everything else whatever.

